# Inflammatory bowel disease and risk for hemorrhoids: a Mendelian randomization analysis

**DOI:** 10.1038/s41598-024-66940-y

**Published:** 2024-07-19

**Authors:** HanYu Wang, Lu Wang, XiaoYu Zeng, ShiPeng Zhang, Yong Huang, QinXiu Zhang

**Affiliations:** 1 Clinical Medical College, Chengdu University of Traditional Chinese Medicine, Chengdu, Sichuan China; 2 Hospital of Chengdu University of Traditional Chinese Medicine, Chengdu, China

**Keywords:** Inflammatory bowel disease, Hemorrhoids, Mendelian randomization, Genetics research, Crohn's disease, Ulcerative colitis

## Abstract

Observational studies have reported an association between inflammatory bowel disease (IBD), which includes Crohn’s disease (CD) and ulcerative colitis (UC), and hemorrhoids (HEM). However, the presence of a causal relationship within this observed association remains to be confirmed. Consequently, we utilized the Mendelian randomization (MR) method to assess the causal effects of IBD on hemorrhoids. We validated the association between IBD and hemorrhoids in humans based on genome-wide association studies (GWAS) data. To investigate the causal relationship between IBD and hemorrhoids, we performed a two-sample Mendelian randomization study using training and validation sets. The genetic variation data for IBD, CD, UC, and hemorrhoids were derived from published genome-wide association studies (GWAS) of individuals of European. Two-sample Mendelian randomization and Multivariable Mendelian randomization (MVMR) were employed to determine the causal relationship between IBD (CD or UC) and hemorrhoids. Genetically predicted overall IBD was positively associated with hemorrhoids risk, with ORs of 1.02 (95% CIs 1.01–1.03, *P* = 4.39 × 10^−4^) and 1.02 (95% CIs 1.01–1.03, *P* = 4.99 × 10^−5^) in the training and validation sets, respectively. Furthermore, we found that CD was positively associated with hemorrhoids risk, with ORs of 1.02 (95% CIs 1.01–1.03, *P* = 4.12 × 10^−6^) and 1.02 (95% CIs 1.01–1.02, *P* = 3.78 × 10^−5^) for CD in the training and validation sets, respectively. In addition, we found that UC in the training set was positively associated with hemorrhoids risk (ORs 1.02, 95% CIs 1.01–1.03, *P* = 4.65 × 10^−3^), while no significant causal relationship between UC and hemorrhoids was shown in the validation set (*P* > 0.05). However, after MVMR adjustment, UC in the training set was not associated with an increased risk of hemorrhoids. Our study showed that there is a causal relationship between CD and hemorrhoids, which may suggest that clinicians need to prevent the occurrence of hemorrhoids in CD patients.

## Introduction

Inflammatory bowel disease (IBD) is a chronic idiopathic disease characterized by intestinal inflammation, encompassing ulcerative colitis (UC) and Crohn’s disease (CD). Recently, IBD has emerged as a global disease with increasing incidence and prevalence^1^. IBD is linked to various complications, including strictures^2^, hemorrhoids^3^ and infections^4^, imposing a significant economic burden on healthcare systems. Studies have indicated that the incidence of symptomatic hemorrhoids in patients with IBD ranges from 3.3 to 20.7%^5–7^.

Hemorrhoids represent a common benign anorectal condition, typically categorized into external hemorrhoids, internal hemorrhoids, and mixed hemorrhoids^8^. The primary clinical manifestations include anal discomfort, pain, bleeding, and other symptoms, making it a major intestinal complication of IBD. When hemorrhoids develop to a point requiring medical intervention, surgical treatment is frequently necessary. In the UK, more than 20,000 hemorrhoids surgeries are performed annually^9^. This undoubtedly imposes a significant medical and health burden. Therefore, for hemorrhoids, prevention holds greater important than treatment. A retrospective study highlighted a 10-year incidence rate of hemorrhagic hemorrhoids in UC patients at 6.7%^10^. Moreover, the prevalence of hemorrhoids in CD patients is approximately 7%^7^. Although prior observational studies have identified a connection between IBD and hemorrhoids, the inherent limitations of observational research preclude definitive conclusions regarding a causal link. Mendelian Randomization, utilizing genetic variation from genome-wide association studies (GWAS), offers a method to explore causal relationships between exposures and outcomes with reduced bias from confounding factors and at a lower cost compared to randomized controlled trials (RCTs). Consequently, we employed MR analysis to investigate the causal relationship between IBD and hemorrhoids.

To our knowledge, there have been no MR studies revealing the causal relationship between IBD and hemorrhoids. In this research, two-sample MR was utilized to examine the causal connection between ethnically diverse IBD (UC or CD) and hemorrhoids, while Multivariate Mendelian randomization (MVMR) was applied to address confounding biases due to shared loci between UC and CD. This investigation offers novel insights into the causal dynamics between IBD (UC or CD) and hemorrhoids.

## Materials and methods

**Figure 1:**
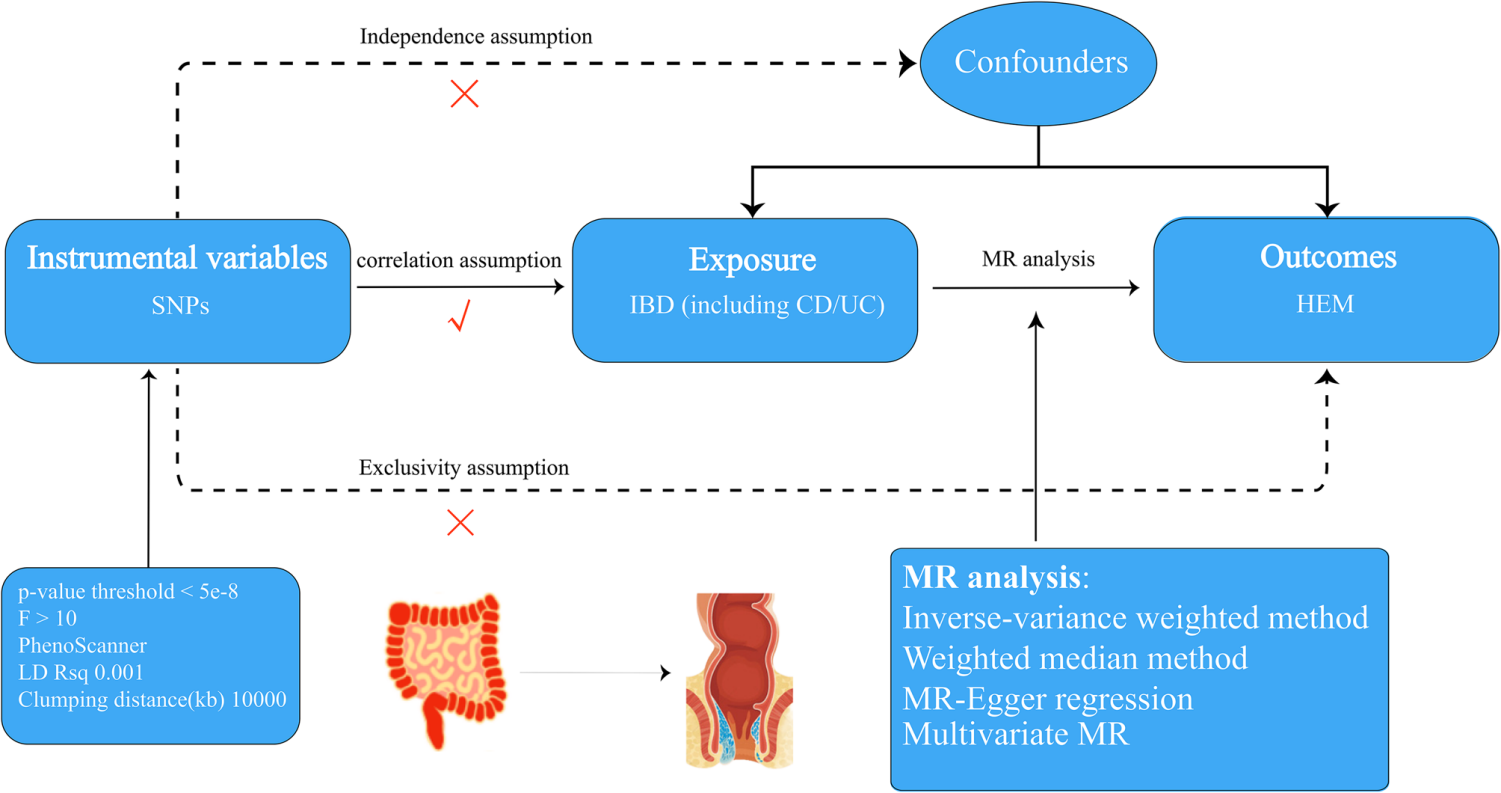
Procedure for an MR analysis of causal associations between inflammatory bowel disease (including Crohn’s disease/Ulcerative colitis) and Hemorrhoids. Checks indicate a correlation between IVs and exposure, while crosses suggest no correlation with confounders or outcomes.

Figure 1 illustrates the concept and analysis process of this study.

### Data sources

The training set was derived from the most extensive genomewide association study (GWAS, https://gwas.mrcieu.ac.uk/), where cohorts included IBD (N = 25,042 cases, 34,915 controls; SNPs = 9,619,016), UC (N = 1,2366 cases, 33,609 controls; SNPs = 9,474,559), CD (N = 12,194 cases, 28,070 controls; SNPs = 9,457,998)^11^. The GWAS data used for validation was from the publicly available International Inflammatory Bowel Disease Genetics Consortium (IIBDGC) pooled studies, including IBD (N = 12,882 cases, 21,770 controls; SNPs = 12,716,084), CD (N = 5,956 cases, 14,927 controls; SNPs = 12,276,506), UC (N = 6,968 cases, 20,464 controls; SNPs = 12,255,197)^12^. Data on hemorrhoid diseases comprised 218,920 patients and 725,213 controls^13^. Table 1 elaborates on the data sources utilized in this study.

**Table 1:** Details of the GWASs included in the Mendelian randomization.

Variable	Category	Number of cases	Number of controls	Data resource	PMID	Year
IBD	Training set	25,024	34,915	ebi-a-GCST004131	28,067,908	2017
CD	Training set	12,194	28,072	ebi-a-GCST004132	28,067,908	2017
UC	Training set	12,366	33,609	ebi-a-GCST004133	28,067,908	2017
IBD	Validation set	12,882	21,770	ieu-a-31	26,192,919	2015
CD	Validation set	5,956	14,927	ieu-a-30	26,192,919	2015
UC	Validation set	6,968	20,464	ieu-a-32	26,192,919	2015
HEM	**–**	218,920	725,213	ebi-a-GCST90014033	33,888,516	2021

### Selection of instrumental variables

In the MR analysis, following established criteria^14–16^, genetic variants were used as instrumental variables (IVs) if they were: (1) strongly associated with the exposure; (2) independent of confounding factors; and (3) established before the MR Analysis. A multistep SNP screening process was conducted to qualify IVs. Initially, IVs for IBD, UC, and CD were selected at a genome-wide significance (GWS) threshold (*P* < 5 × 10^−8^)^17^, while for hemorrhoids, a suggestive threshold (*P* < 5 × 10^−5^) was adopted to ensure adequate study power. Additionally, we set the criteria as r^2^ = 0.001 and the width of the linkage disequilibrium (LD) region = 10,000 kb to avoid LD^18^. Lastly, F statistics were used to confirm a strong association between the IVs and exposure, with F-statistics greater than 10 being widely accepted as indicative of a strong correlation^19^. The equation for the F-value used in this study is F = Beta^2^/Se^2^, where Beta is the allele effect value, and Se is the estimated standard error of Beta. Furthermore, SNPs fulfilling these criteria were verified against the Phenoscanner database (www.phenoscanner.medschl.cam.ac.uk) to check for the presence of a secondary phenotype that could introduce bias^20^.

### MR analyses

Before commencing the analysis, we first synchronized exposure and outcome data to ensure alignment of effect alleles with the positive strand, excluding intermediate allele frequency palindromes from further MR analysis^17^. The inverse variance weighting (IVW) method served as the primary approach for estimating causal effects. Within the two-sample MR framework, IVW stands as the most robust technique for establishing causality^21^. Results were further supported by MR-Egger and weighted median analyses, providing a comprehensive validation when findings across these three models aligned. To affirm the integrity of our outcomes, we undertook separate evaluations for heterogeneity and multiplicity. Heterogeneity within the IVW model was gauged using Cochran’s Q-test^22^, with a *P* < 0.05 indicating significant heterogeneity. Notably, heterogeneity does not inherently compromise the IVW model’s validity, especially when employing a random-effects model in MR analysis^23^. Considering the influence of unknown confounders on genetic diversity and causal effects, we used MR–Egger regression to assess whether the included SNPs were potentially horizontally pleiotropic, and horizontally pleiotropic results (*P* < 0.05) were excluded^24^. In addition, we used the Mendelian randomization pleiotropy residual sum and outlier (MR-PRESSO) algorithm to identify and remove any outliers with significant differences. After excluding outliers, the MR analysis was repeated, and the causal effect was reassessed^25^. Furthermore, a leave-one-out analysis was conducted to determine if omitting a single SNP significantly altered the results. Finally, MVMR analysis was applied to the positive MR findings of two samples to rectify biases induced by the causal linkage of multiple exposure factors through genetic instruments on the same outcome variable.

### Statistics analysis and visualization

This investigation represents a secondary analysis of previously published data, with no modifications to the original dataset. All analytical processes were executed using R software (version 4.3.1). The “TwoSampleMR” and “MRPRESSO” packages facilitated the MR analysis, while the “forest plot” packages were employed for graphical representations.

## Results

### Two-sample MR analysis for the causal association between IBD/CD/UC and hemorrhoids

There were 117 SNPs (IBD), 89 SNPs (CD) and 62 SNPs (UC) with *P* < 5 × 10^−8^. Following the exclusion of intermediate allele frequency palindromes and unmatched SNPs, 97, 76, and 51 IVs respectively were retained for MR analysis. Additionally, the MR-PRESSO test identified outlier IVs as follows: for IBD to hemorrhoids, rs56062135 and rs11066188; for CD to hemorrhoids, rs4316387, rs6808936, rs72743461; and for UC to hemorrhoids: rs56062135, rs72704802, rs7523335, rs9260809, rs989960. These outlier IVs were removed and the analysis was conducted again. Despite these adjustments, the outcomes remained consistent (Supplementary Table 1 provides detailed genetic variation information).

**Figure 2:**
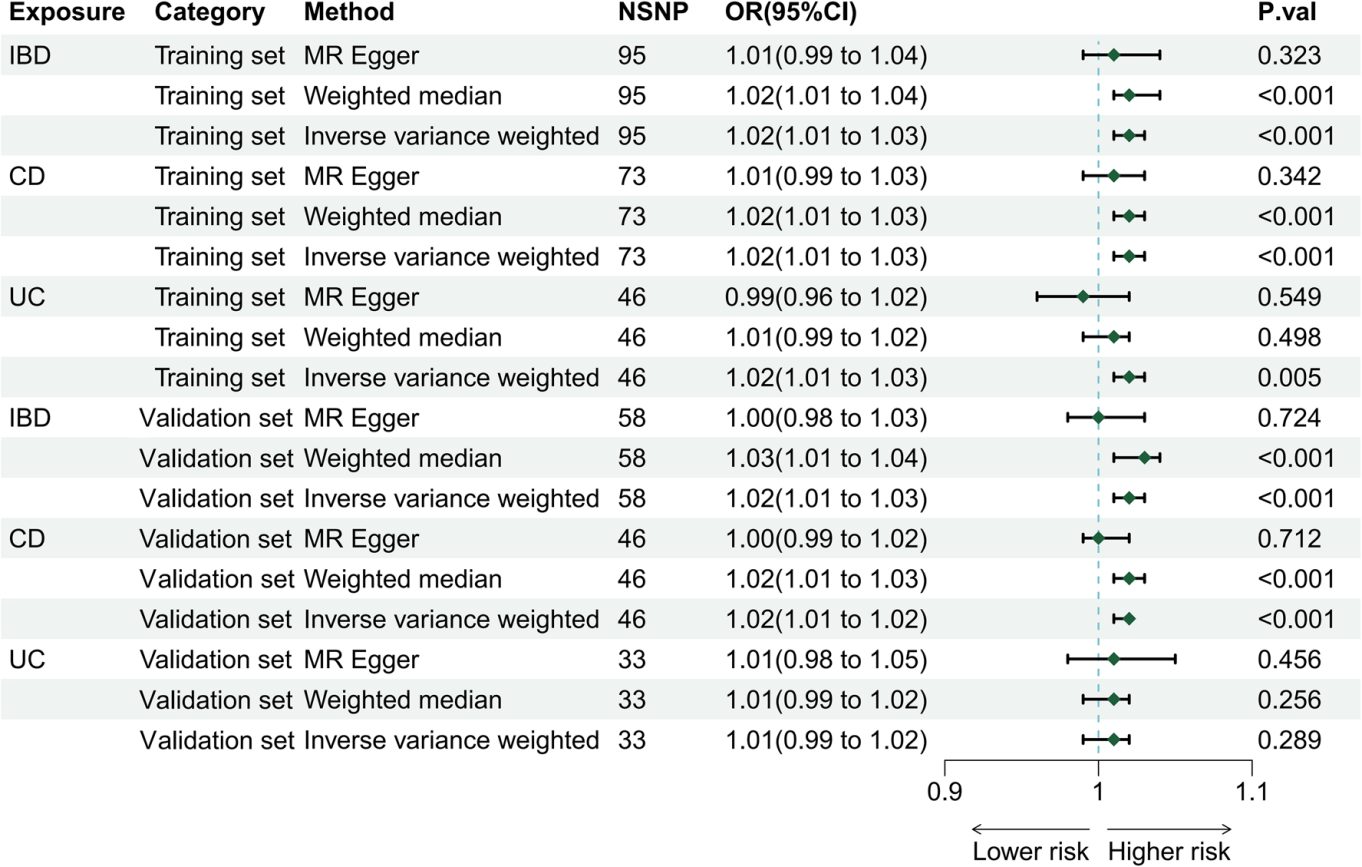
The risk association between IBD (including CD/UC) and hemorrhoids in a forest plot. (IBD: Inflammatory bowel disease; CD: Crohn’s disease; UC: Ulcerative colitis; NSNP: the number of single nucleotide polymorphisms used in MR analysis; OR: odds ratio; CI: confidence interval).

The causal link between IBD/CD/UC and hemorrhoids was assessed using three methods (MR Egger, Weighted median, IVW), yielding the following ORs: For IBD and hemorrhoids, ORs were 1.01 (95% CIs 0.99, 1.04; *P* = 3.23 × 10^−1^), 1.02 (95% CIs 1.01, 1.04; *P* = 4.30 × 10^−4^), 1.02 (95% CIs 1.01, 1.03; *P* = 4.39 × 10^−4^). For CD and hemorrhoids, ORs were 1.01 (95% CIs 0.99, 1.03; *P* = 3.42 × 10^−1^), 1.02 (95% CIs 1.01, 1.03; *P* = 4.86 × 10^−4^), 1.02 (95% CIs 1.01, 1.03; *P* = 4.12 × 10^−6^). For UC and hemorrhoids, ORs were 0.99 (95% CIs 0.96, 1.02; *P* = 5.49 × 10^−1^), 1.01 (95% CIs 0.99, 1.02; *P* = 4.98 × 10^−1^), 1.02 (95% CIs 1.01, 1.03; *P* = 4.65 × 10^−3^) (Fig. 2). Collectively, these findings indicate that IBD and CD are associated with an increased risk of hemorrhoids from both epidemiological and genetic perspectives. (Supplementary Table 3 provides detailed information).

**Figure 3:**
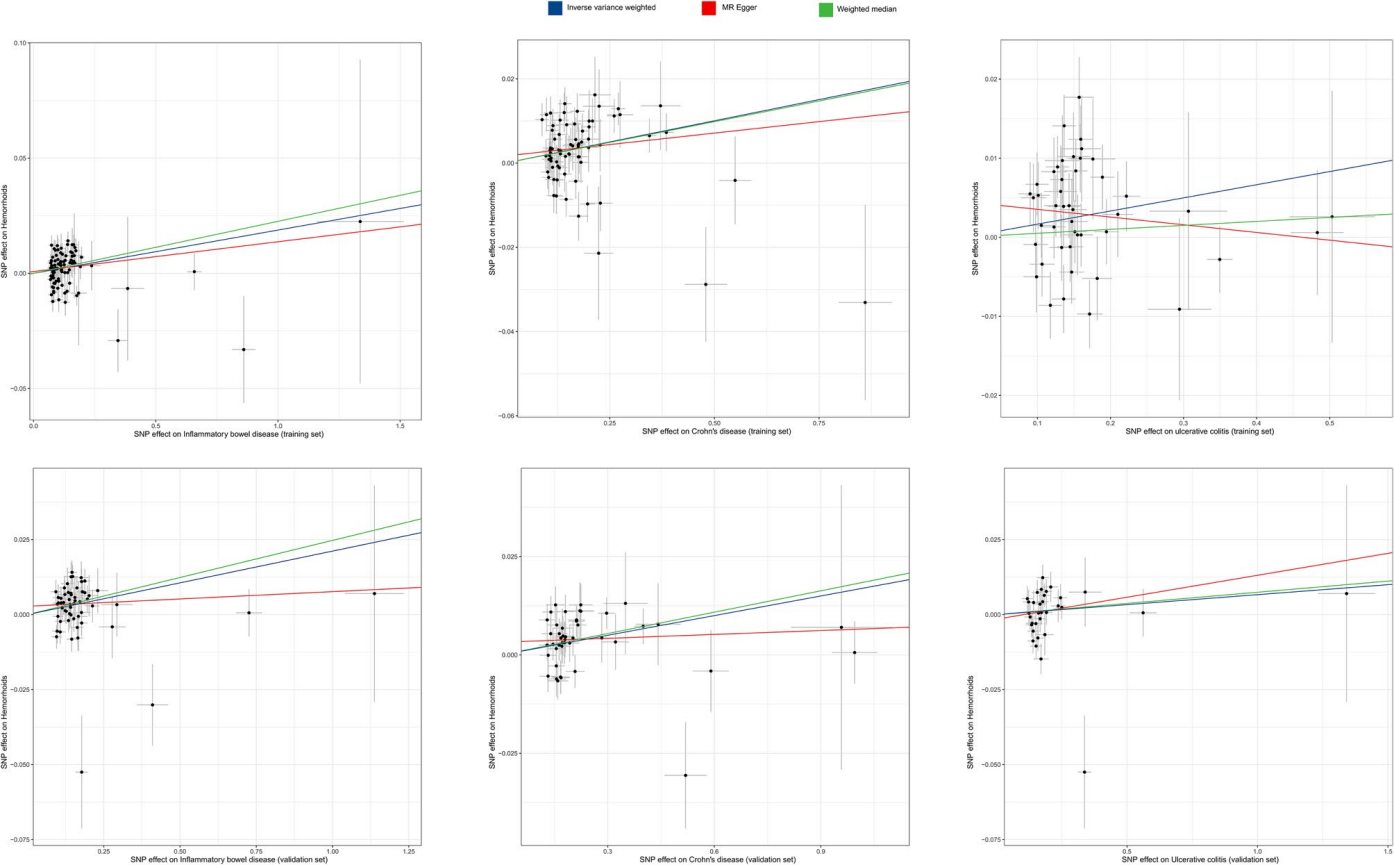
Scatter plot of MR analyses from inflammatory bowel disease (including Crohn’s disease/Ulcerative colitis) to Hemorrhoids. SNP: Single Nucleotide Polymorphism. The slope of each line represents the effect estimation of different methods using MR.

The scatter plot (Fig. 3) displays the estimated effect sizes of genetically predicted IBD, UC, and CD on hemorrhoids. Despite the presence of heterogeneity in the Cochran Q test results, the heterogeneity was deemed acceptable when employing the random effects IVW analysis as the principal method^23^. The p values of the MR-Egger intercept exceeded 0.05 across the board, suggesting an absence of pleiotropic interference. Additionally, neither leave-one-out analysis nor the funnel plot (Supplementary Fig. 1) identified any outlier IVs, further affirming the robustness of our findings.

We selected an IBD dataset from different databases as the validation set. After removing pleiotropic SNPs, we obtained 58 SNPs for IBD, 46 for CD, and 33 for UC, all of which were robust instrumental variables. The results of MR Egger regression suggested that genetic pleiotropy did not introduce any bias (*P* > 0.05). The heterogeneity test by IVW method showed that there was heterogeneity among genes, so random effects IVW analysis was used as the main analysis method.

The causal link between IBD/CD/UC and hemorrhoids was assessed using three methods (MR Egger, Weighted median, IVW), yielding the following ORs: For IBD and hemorrhoids, ORs were 1.00 (95% CIs 0.98, 1.03; *P* = 7.24 × 10^−1^), 1.03 (95% CIs 1.01, 1.04; *P* = 7.81 × 10^−5^), 1.02 (95% CIs 1.01, 1.03; *P* = 4.99 × 10^−5^). For CD and hemorrhoids, ORs were 1.00 (95% CIs 0.99, 1.02; *P* = 7.12 × 10^−1^), 1.02 (95% CIs 1.01, 1.03; *P* = 7.46 × 10^−4^), 1.02 (95% CIs 1.01, 1.02; *P* = 3.78 × 10^−5^). For UC and hemorrhoids, ORs were 1.01 (95% CIs 0.98, 1.05; *P* = 4.56 × 10^−1^), 1.01 (95% CIs 0.99, 1.02; *P* = 2.56 × 10^−1^), 1.01 (95% CIs 0.99, 1.02; *P* = 2.89 × 10^−1^) (Fig. 2). Overall, these results showed that both total IBD and CD could increase the risk of hemorrhoids from a genetic perspective. (Supplementary Table 3 provides detailed information). Additionally, neither leave-one-out analysis nor the funnel plot (Supplementary Fig. 2) identified any outlier IVs, further affirming the robustness of our findings.

### Multivariate MR

MVMR facilitates the examination of causality between multiple exposure factors, as influenced by genetic instruments, on a singular outcome variable, with the potential to minimize bias stemming from confounding factors^26^. To address the confounding elements associated with CD and UC, we executed a multivariate MR analysis of the relationship between UC and CD to hemorrhoids in the training set, corroborating our initial observations. The analysis demonstrated a significant causal link between CD and hemorrhoids (*P* = 0.03), whereas no causal association was identified between UC and hemorrhoids (*P* = 0.33), aligning with the outcomes from our two-sample MR analysis. The detailed findings of the MVMR are documented in Supplementary Table 2.

## Discussion

To the best of our knowledge, this study is the inaugural MR investigation to explore the causal relationship between IBD and hemorrhoids. Our results confirmed a significant positive relationship between CD and hemorrhoids. In addition, although MR results showed that UC could also increase the incidence of hemorrhoids, it was considered that most IBD-related susceptibility gene loci were simultaneously associated with UC and CD^27^. Therefore, the causal relationship between UC and hemorrhoids may be confused by CD. After eliminating the mixing of CD and UC by MVMR, we found that UC did not increase the risk of hemorrhoids.

The pathogenesis of IBD is likely interconnected with immune system dysregulation, Genetic inheritance, alterations in intestinal microbiota, and micronutrient malabsorption, among other factors. Hemorrhoids rank as one of the most prevalent anorectal disorders, with incidence rates ranging from 4.4 to 45 percent^28^. Personal hygiene, lifestyle habits, dietary habits, physical differences, genetic factors, and immune factors can all induce the formation of hemorrhoids. According to the National Institute of Diabetes and Digestive and Kidney Diseases (NIDDK), approximately half of American adults experience symptoms or signs of hemorrhoids at some juncture. As a frequent complication of IBD, hemorrhoids can exacerbate the primary disease and detrimentally affect patient quality of life. Particularly, severe hemorrhoids may lead to intestinal damage, necessitating surgical intervention and complicating IBD management.

The longstanding theory of varicose veins posits that hemorrhoids are associated with varicosities in the anal canal. Yet, this hypothesis does not fully account for hemorrhoids formation, and evidence suggests that patients with portal hypertension do not exhibit an increased hemorrhoids incidence^29^. Currently, the widely accepted theory involves the downward displacement of the anal cushion^30^. Comprising mucous membranes, blood vessels, smooth muscle, elastic fibers, and other submucosal structures, the anal cushion may protrude into the anal canal to form hemorrhoids due to hypertrophy or the degradation/deterioration of its supportive tissue. Currently, various enzymes and mediators implicated in the degradation of supportive tissue in the anal pad have been identified.

Some studies have found that miRNAs play a key role in the pathogenesis of IBD and hemorrhoids^31–34^. miRNAs are a group of small non-coding RNAs, approximately 18–22 nucleotides in length^35^, that function as regulators of post-transcriptional gene expression and circulate in human peripheral blood in a stable form^35,36^. miRNA expression is critical in a variety of human diseases, including cancer, autoimmune disorders, cardiovascular diseases, and neurodegenerative conditions. miRNAs are involved in the onset and development of diseases, with some being pathologically specific. Some studies have found that the serum samples of IBD patients exhibit higher levels of miR-16, miR-21, and miR-223 compared to control groups, and the levels of miR-16, miR-21, and miR-223 in patients with CD were higher than those in patients with UC^31,32^. There is evidence that the abnormal expression of miR-21 and miR-223 can induce the production and upregulation of matrix metalloproteinase-9 (MMP-9)^37–40^. Matrix metalloproteinases (MMPs) are a family of proteolytic enzymes that contain active zinc ions and are capable of degrading extracellular matrix components, including elastin, fibronectin, and collagen^41^. Additionally, miR-21 has been found to be expressed in immune cells and promote the production of inflammatory cytokines such as TNF-α, IFN-γ, and IL-1β, which are closely related to the pathogenesis of IBD and can induce the production of MMPs^42–44^. The overexpression of MMPs can degrade extracellular proteins^29^, leading to tissue destruction and the subsequent breakdown of the supportive tissue of the anal pad. This process facilitates the downward displacement of the anal pad, ultimately resulting in the formation of hemorrhoids^45–47^. Research by Raffaele Serra et al. demonstrated a significant increase in MMP levels in patients with hemorrhoids^48^. Additionally, a study by Wei Han et al. revealed the overexpression of MMP-9 in hemorrhoids, suggesting its association with the breakdown of elastic fibers^49^. Therefore, we speculate that the occurrence of hemorrhoids in patients with IBD may be related to the activation of MMPs. However, within the field of epigenetic regulation, there are relatively few studies on the regulatory mechanisms of non-coding RNA (especially miRNAs) in the development of hemorrhoids. We look forward to future research to elucidate the relationship and potential mechanisms underlying these findings.

In addition, research indicates that most IBD patients experience changes in bowel habits, such as abdominal diarrhea and a sense of urgency to defecate^50,51^. Diarrhea was identified as a common risk factor for hemorrhoids in a case–control study^52^. We believe that loose or frequent bowel movements in IBD patients may cause the anal sphincter to contract for extended periods to maintain stool control. Chronic contraction of the anal sphincter increases anal pressure, impeding venous outflow from the hemorrhoid pad. Since arterial inflow is not affected by increased sphincter pressure, reduced venous outflow causes the hemorrhoid pad to enlarge, potentially leading to bleeding and prolapse. Moreover, several studies have emphasized the crucial role of the gut microbiome in maintaining gut homeostasis^53^. Disruption in the interaction between microbiota and the immune system can lead to bowel inflammation. For instance, IBD is characterized by alterations in the equilibrium of microbial populations^54^. Changes in the composition or diversity of intestinal microbial communities may influence the pathogenesis of hemorrhoids^55,56^. We suspect that gut microbiome alterations caused by IBD may also contribute to the development of hemorrhoids in IBD patients. However, due to the complexity of the gut microbiome as an ecosystem, regulatory networks and confounding factors among various bacteria types exist. Further experimental studies are necessary to elucidate the mechanisms linking the gut microbiome with both IBD and hemorrhoids.

In conclusion, high-quality evidence substantiating this hypothetical mechanism linking IBD to hemorrhoids remains elusive. Future animal experiments and clinical investigations are anticipated to validate and enrich these hypotheses.

It is important to highlight that currently, there is no universally accepted approach to treating hemorrhoids in patients with IBD. Some experts argue that surgical intervention for hemorrhoids in IBD patients ought to be highly restricted due to the elevated risk of post-hemorrhoidectomy complications compared to the general population, with the potential for severe adverse outcomes. Jeffery et al. were among the first to highlight the significant risk of serious complications following hemorrhoidectomy in CD patients. In their study, 6 out of 20 CD patients who underwent hemorrhoidectomy experienced severe complications necessitating rectal resection^57^. Consequently, for IBD patients suffering from hemorrhoids, pharmaceutical management should be prioritized, and surgical options should be cautiously considered only when conservative treatments prove ineffective, given the high risk of severe postoperative complications^3^. Nonetheless, some researchers maintain that surgical treatment remains a viable option provided there is no active IBD^28,58,59^. Present findings indicate that the complication rate post-hemorrhoidectomy in CD patients is nearly threefold higher than in UC patients (17.1% vs 5.5%)^60^. In Jeffery et al.’s investigation, among 24 UC patients subjected to hemorrhoid surgery, only one developed a postoperative anal fistula requiring rectal resection^57^. Our MR analyses lend further support to the causal link between CD and hemorrhoids specifically in European populations. Medical practitioners should be cognizant of the potential for infection, malignant complications, and escalated healthcare costs following hemorrhoidectomy in IBD patients. Thus, proactive prevention, comprehensive medical management, and a multidisciplinary approach are essential for addressing hemorrhoids in CD patients.

The strength of this study lies firstly in its novel approach; as there are no randomized controlled trials (RCTs) exploring the link between IBD and hemorrhoids, and given the inevitable confounders present in existing clinical observations, utilizing large-scale GWAS data for MR analysis allowed for a more precise causal assessment, confirming a causal link between IBD and hemorrhoids within the European population. Additionally, by employing MVMR analysis, the hybrid deviation between UC and CD was addressed and corrected.

However, our study is not without its limitations. Primarily, due to the constraints of GWAS datasets, future foundational research is required to determine causality and explore potential pathogenic processes, which are crucial for clinical intervention. Also, while the data relate to IBD, they lack specificity regarding the frequency and severity of IBD episodes. Further investigation is necessary to determine the connection between IBD severity and the risk of developing hemorrhoids. In addition, Our GWAS data only used the European sample database, thereby limiting the universality and applicability of the findings to other populations. The incidence and prevalence of IBD are different in different regions and countries. Therefore, we are optimistic about the prospect of future large-scale cross-regional and ethnic longitudinal studies that will offer a dynamic view on how the risk of hemorrhoids evolves over time among IBD patients across different geographical and ethnic backgrounds.

## Conclusion

In conclusion, our research established a genetic causal link between IBD and hemorrhoids within European demographics, predominantly influenced by CD. This investigation not only corroborates the causal relationship between IBD and hemorrhoids, but also unveils new insights into the potential mechanisms underpinning IBD and hemorrhoids associations. Such findings promote the generation of novel hypotheses concerning the pathogenesis of hemorrhoids. The advancements achieved through this study provide a solid groundwork for future endeavors in validating intervention trials, identifying new therapeutic targets, and informing the development of pharmacological interventions.

### Supplementary Information

Supplementary Figure 1.

Supplementary Figure 2.

Supplementary Table 1.

Supplementary Table 2.

Supplementary Table 3.

## Data Availability

All data used in the current study are publicly available GWAS summary data (GWAS, https://gwas.mrcieu.ac.uk/).
