# Supplementary material for: Inflammatory bowel disease and risk for hemorrhoids: a Mendelian randomization analysis
Source: Sci Rep. 2024 Jul 19;14:16677. doi: 10.1038/s41598-024-66940-y (PMC11271563; doi:10.1038/s41598-024-66940-y)
Supplement: Supplementary file 1 — Supplementary Figure 1. [file 41598_2024_66940_MOESM1_ESM.docx]

1. Leave-one-out plot and Funnel plot of MR analyses from Inflammatory Bowel Disease to Hemorrhoids of the training set.

1. Leave-one-out plot and Funnel plot of MR analyses from Crohn's disease to Hemorrhoids of the training set.

**
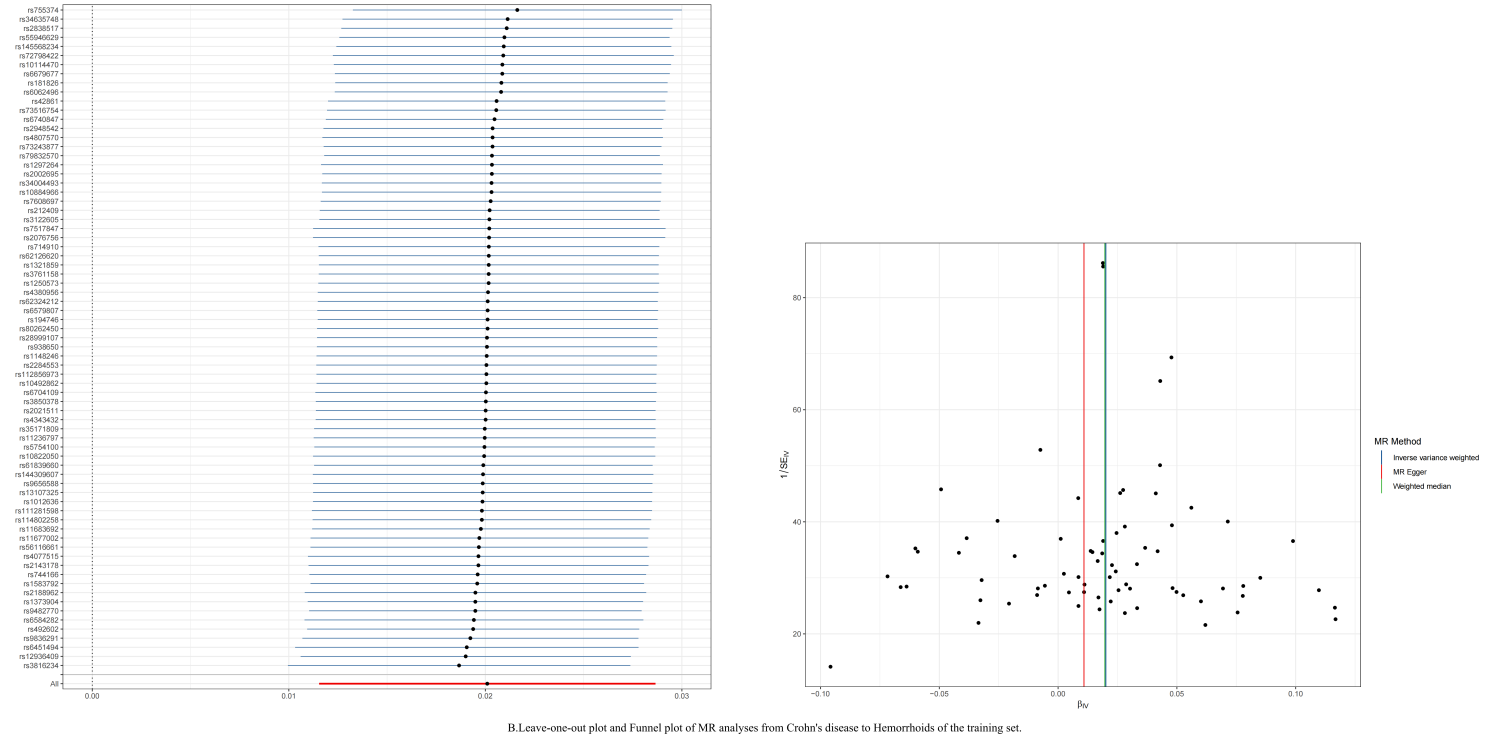
**

1. Leave-one-out plot and Funnel plot of MR analyses from Ulcerative colitis to Hemorrhoids of the training set.
