# Supplementary material for: Inflammatory bowel disease and risk for hemorrhoids: a Mendelian randomization analysis
Source: Sci Rep. 2024 Jul 19;14:16677. doi: 10.1038/s41598-024-66940-y (PMC11271563; doi:10.1038/s41598-024-66940-y)

1. Leave-one-out plot and Funnel plot of MR analyses from Inflammatory Bowel Disease to Hemorrhoids of the validation set.

1. Leave-one-out plot and Funnel plot of MR analyses from Crohn's disease to Hemorrhoids of the validation set.

1. Leave-one-out plot and Funnel plot of MR analyses from Ulcerative colitis to Hemorrhoids of the validation set.


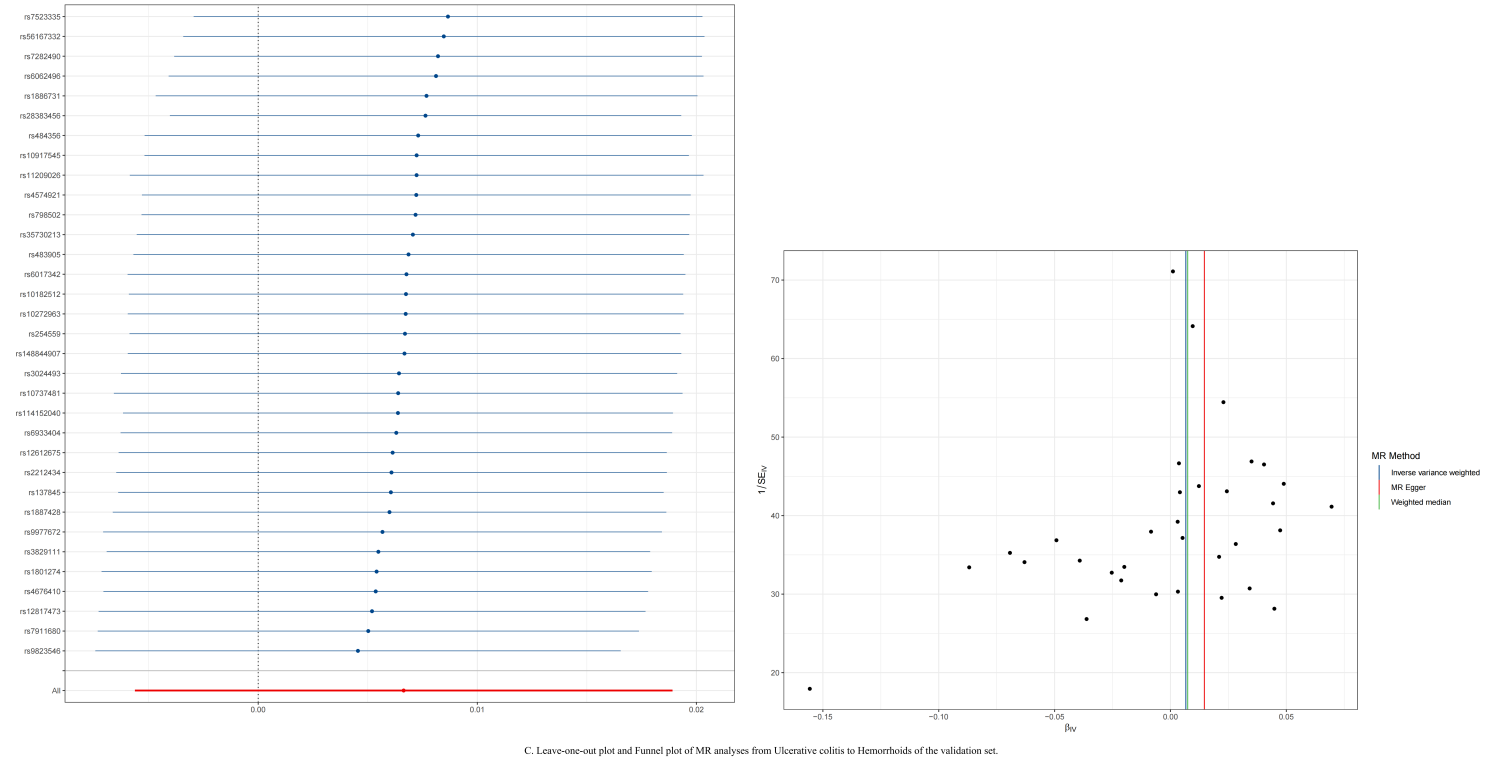

Supplement: Supplementary file 2 — Supplementary Figure 2. [file 41598_2024_66940_MOESM2_ESM.docx]
